# Trop2 Forms a Stable Dimer with Significant Structural Differences within the Membrane-Distal Region as Compared to EpCAM

**DOI:** 10.3390/ijms221910640

**Published:** 2021-09-30

**Authors:** Miha Pavšič

**Affiliations:** Department of Chemistry and Biochemistry, Faculty of Chemistry and Chemical Technology, University of Ljubljana, Večna Pot 113, SI-1000 Ljubljana, Slovenia; miha.pavsic@fkkt.uni-lj.si; Tel.: +386-1-479-8550

**Keywords:** Trop2, extracellular part, ectodomain, Trop2EX, crystal structure, dimer, EpCAM

## Abstract

Trop2 is a cell-surface transmembrane glycoprotein involved in the maintenance of epithelial tissue integrity and is an important carcinoma marker. It shares similar claudin-interaction capacity with its paralogue EpCAM, and both are implicated in signaling triggered by proteolytic cleavage within the ectodomain. However, the cell proliferation-regulating interactions with IGF-1, neuregulin-1, and α_5_β_1_ integrin appear to be Trop2-specific. To illuminate the structural differences between Trop2 and EpCAM, we report the first crystal structure of a Trop2 ectodomain dimer and compare it to the analogous part of EpCAM. While the overall fold of the two proteins is similar, the dimers differ. In Trop2, the inter-subunit contacts are more extensive than in EpCAM, and there are two major differences in the membrane-distal regions. The immunogenic N-terminal domain is in Trop2 almost colinear with the dimer interface plain and consequently more laterally exposed, and the cleft of yet unknown functionality between the two subunits is almost absent. Furthermore, the site of initial signaling-associated proteolytic cleavage in Trop2 is accessible in the dimeric state, while in EpCAM dimer destabilization is required. The structural differences highlight the divergent evolutionary path of the two proteins and pave the way for their structure-based utilization in therapy.

## 1. Introduction

Trop2 (trophoblast cell surface antigen 2) is cell-surface transmembrane type-1 glycoprotein expressed in normal epithelial cells of various tissues [1]. It is also a stem/progenitor cell marker [2,3], and is frequently overexpressed in carcinomas (recently reviewed by Lenárt and coworkers [4]). It is evolutionarily related to another carcinoma marker with a partially overlapping expression pattern—EpCAM. Both are promising targets in carcinoma diagnosis, prognosis, and therapeutic approaches [4,5], which calls for a detailed functional and structural comparison. While EpCAM has already been structurally characterized [6,7,8], no equivalent structural data have been available for Trop2 until now.

**Figure 1:**
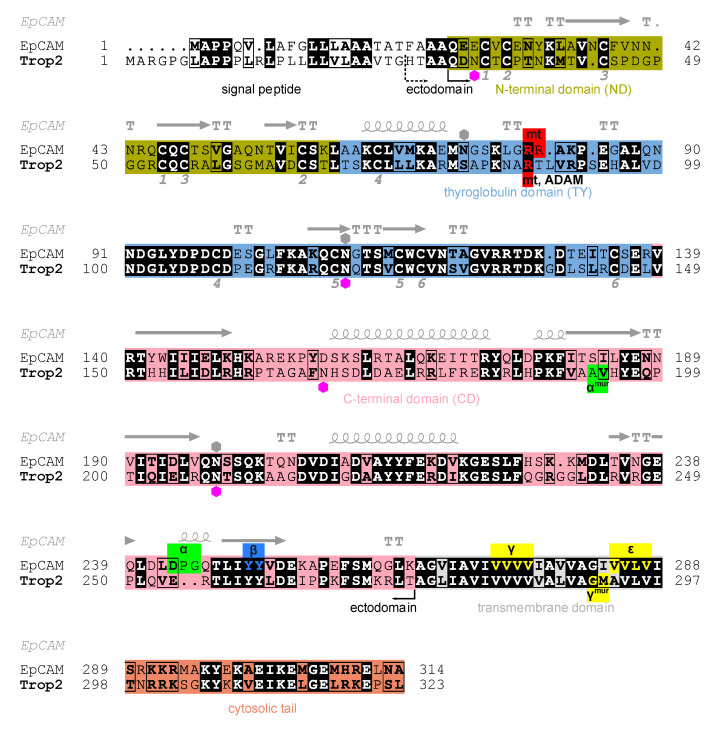
Alignment of human Trop2 (UniProt P09758-1) and EpCAM (UniProt P16422-1) amino acid sequences with marked N-terminal (olive), thyroglobulin (light blue), C-terminal (pink), transmembrane (grey) and cytosolic (orange) regions/domains. Identical residues are shown in bold and with black background, and chemically similar residues in bold and in rectangles. Hexagons denote N-glycosylation sites as annotated in UniProt for Trop2 (magenta) and EpCAM (grey). Pairs of cysteine residues forming a disulfide bridge are denoted by equal numbers below the alignment (from one to six, corresponding to six disulfide bridges). Secondary structure elements in EpCAM (PDB ID 4MZV [6]) are shown above EpCAM sequence: α-helices (spiral), β-strands (arrows), α-turns (TTT) and β-turns (TT). Smaller red, green, blue and yellow boxes denote experimentally determined proteolytic cleavage sites on human Trop2 and EpCAM, by matriptase (mt), TACE (α), ADAM10 (ADAM), β-secretase 1 (BACE, β) and γ-secretase (γ, ε). Cleavage sites determined using a murine protein are denoted by “mur” superscript; the corresponding residues in human protein were deduced from alignment of murine and human sequences. Two alternative N-termini of the ectodomain are marked: H27 (currently annotated in UniProt) and Q31 (predicted using SignalP [25]). Figure was prepared using ESPript 3.0 [26], and the output has been manually edited to include features and topological regions.

The intronless Trop2 gene (*TACSTD2*) emerged after a retroposition event from the EpCAM gene (*TACSTD1*) [9]. Trop2 and EpCAM are, in terms of amino acid sequence identity, very similar (48%, Figure 1). They are composed of three topological regions: ectodomain representing the largest portion of the protein, a single transmembrane region, and a short cytosolic tail (Figure 1). The crystal structure of EpCAM ectodomain revealed a compact subunit composed of three domains: N-terminal (ND), thyroglobulin type-1 (TY), and C-terminal domain (CD) [6]. The protein crystallized as a dimer which was later shown to be a biologically relevant cell-surface form [10]. A dimeric form has also been demonstrated for Trop2 ectodomain in solution [11]. Dimerization has implications in EpCAM signaling function which involves sequential proteolytic cleavages [12,13], and at least partial opening of the ectodomain dimer is required to reveal the α-site for initial cleavage by tumor necrosis factor-α-converting enzyme (TACE) [8,13,14]. Analogous proteolytic cleavages were also demonstrated for Trop2 where cleavage at the α-site by TACE (also known as a disintegrin and metalloproteinase (ADAM) 17) within the ectodomain is followed by intramembrane cleavage by the γ-secretase complex [15]. The released cytosolic tail of Trop2 (Trop2IC) participates in signaling via β-catenin [15], similarly as in EpCAM [16], and Trop2IC–β-catenin interaction is also linked to induction of epithelial-to-mesenchymal transition (EMT) in cancer [17]. Unlike as in EpCAM, the Trop2 cytosolic tail is also a potential target for phosphorylation [18] which triggers a significant structural change of this short region [19]. Another difference is the position of the cleavage sites within ectodomains od Trop2 and EpCAM: α-sites in the two proteins are separated by nine amino acid residues, and for Trop2 cleavage at β-site has not been demonstrated (Figure 1) [15]. Matriptase is another enzyme that cleaves EpCAM and Trop2. Cleavage within the TY domain breaks their interaction with claudins and has, consequently, implications in claudin trafficking and epithelial integrity [20]. While the absence of functional EpCAM results in the loss of intestinal tissue integrity [21,22], Trop2 seems to be dispensable, however it is able to prevent the development of severe congenital tufting enteropathy (CTE) [23]. In Trop2 the matriptase cleavage site is also targeted by another sheddase, ADAM10, and the cleavage is associated with enhanced metastatic potential [24]. Trop2 is also involved in signaling via other pathways which has been recently reviewed by Lenárt and coworkers [4].

Besides different position of some of the sites involved in signaling-associated proteolytic processing, Trop2 and EpCAM have different interaction partners which is connected to their different role in regulating cell proliferation. First, EpCAM ectodomain directly interacts with epidermal growth factor receptor (EGFR) and induces proliferation while counteracting EMT [27]; for Trop2 such interaction has not (yet) been demonstrated. On the other hand, there are several reports of Trop2-specific interactions. First, Trop2 inhibits signaling via insulin-like growth factor 1 receptor (IGF-1R) and anaplastic lymphoma kinase (ALK), possibly via direct interaction of Trop2 ectodomain with their cognate ligands (IGF-1 and midkine (MDK), respectively) [28,29]. Second, Trop2 ectodomain interacts with neuregulin-1 and thereby negatively affects ErbB3 activation [30]. Third, Trop2 also interacts with α_5_β_1_ integrin (interaction via ectodomains), displaces focal adhesion kinase from focal contacts, and promotes cancer cell migration [31,32]. Structural details regarding specific interaction regions are yet unknown. Therefore, considering only partial similarity in proteolytic processing and significantly different interaction partners, Trop2 and EpCAM represent a unique case of evolutionary divergence which requires detailed structural addressing.

Here we report the first crystal structure of human Trop2 ectodomain (Trop2EX) and provide a detailed structural comparison with EpCAM, also considering the functional differences between the two proteins. We show that while the overall structure and dimeric assembly of the two proteins is very similar, there are considerable structural differences, particularly in the membrane-distal region.

## 2. Results and Discussion

### 2.1. Protein Sample Preparation for Crystallization

For crystallization a mutant variant of Trop2 ectodomain was used where two putative N-glycosylation sites were mutated (N120Q, N208Q) while the two other sites were left intact (N33, N168) to achieve adequate balance between protein sample homogeneity and solubility. The glycosylation level of the recombinant protein was low (less than 5 kDa; Figure S1a), and the N-terminal glutamine residue (Q31) was modified to pyroglutamate (Figures S2 and S3), similarly as in EpCAM [6,33]. This indicates that mature Trop2, at least if produced in insect cells, starts with pyroGlu31 and not with H27 as previously expected (Figure 1), which is in line with SignalP prediction (Figure S4).

### 2.2. Trop2 Ectodomain Structure Overview and Molecular Assemblies

#### 2.2.1. Structure Determination

The crystal structure of Trop2EX (N120Q, N208Q) was solved using X-ray diffraction data collected from a cryo-preserved crystal (Figure S1b). Four copies of Trop2 ectodomain were located within the asymmetric unit (chains A to D) which form various assemblies as discussed in the next section. Statistics on data collection and structure refinement are collected in Table 1.

#### 2.2.2. Molecular Assemblies

Molecules within the crystal form an extensive network of intermolecular interactions. Analysis of these crystal contacts can be very informative in identification of biologically relevant molecular assemblies [35,36,37], as in the case of crystal structure of EpCAM were an ectodomain dimer formed by subunits from adjacent asymmetric units was identified [6] and later confirmed as a biologically relevant assembly [10].

**Figure 2:**
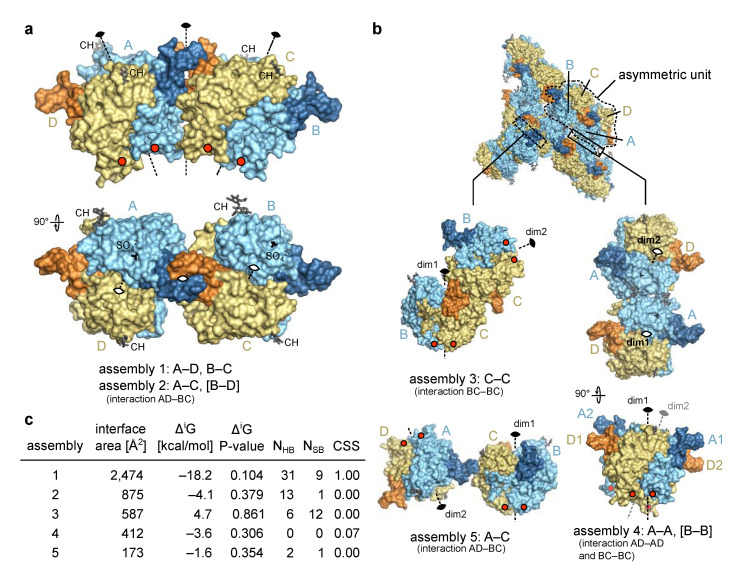
Molecular assemblies within the asymmetric unit and beyond. (**a**) In asymmetric unit, four polypeptide chains (chains A to D) corresponding to four copies of Trop2 ectodomain were found and are shown here as molecular surface. Chains A and B are shown in pale cyan and their N-terminal domains in dark blue, while chains C and D are shown in light yellow and their N-terminal domains in pale orange. Improper rotational (non-crystallographic) symmetry axes are shown as dashed lines and denoted with the standard 2-fold symmetry symbol. Sulfate ions (SO_4_^2−^, only in chains A and B) and N-linked carbohydrates (CH) are shown as black and dark grey sticks, respectively. Red dots indicate positions of C-termini. Interaction in square brackets is not shown, however it is implied by symmetry. (**b**) Alternative assemblies as devised from crystal contacts and with interface area larger than 150 Å^2^. Color-coding is the same as in (**a**). Dim1 and dim2 denote two-fold axis of each dimer. Interaction in square brackets is not shown, however it is implied by symmetry. (**c**) Table of assemblies and associated interface area, Δ^i^G and associated P-value, number of hydrogen bonds (N_HB_) and salt bridges (N_SB_) at the interface, and CSS as reported by PISA [38]. In case of two equivalent interfaces average values are reported, for example as in assembly 4.

The four chains in the asymmetric unit of the Trop2 ectodomain crystal form an elongated assembly (Figure 2a) with three two-fold rotational non-crystallographic symmetry (NCS) axes. The middle one of the three axes, located at the contact of NDs (from chains A and C, forming assembly 2), operates between two tighter smaller assemblies AD and BC, each of them with its own two-fold rotational axis (assembly 1). Three additional assemblies with an interface area larger than 150 Å^2^ were identified by analysis of crystal contacts (Figure 2b). Of these five assemblies, only assembly 1 has a very large interface area of 2474 Å^2^ (average of interfaces between AD and BC) (Figure 2c). At the same time, this assembly has the most negative solvation free energy gain upon interaction (Δ^i^G) and the lowest P-value indicating a more hydrophobic interaction, generally connected with higher interaction specificity [38]. This interaction involves 31 hydrogen bonds and 9 salt bridges (average of interfaces AD and BC), and the highest possible complexation significance score (CSS) of 1 in interface analysis indicates its very high relevance in the formation of the complex. Other assemblies (2 though 5) have a much higher P-value and a CSS of approximately 0, indicating that the assembly 1 is significantly more relevant and stable in solution.

The existence of a Trop2 ectodomain dimer has been demonstrated in solution by chemical cross-linking and size exclusion chromatography coupled to multi-angle laser light scattering [11]. Considering the interface analysis of the identified assemblies (above) it is very likely that the dimer identified in solution corresponds to the assembly 1 (2 variants, AD and BC). Even more, this assembly is very similar to the dimer of EpCAM ectodomains where the C-termini are located at the same side of the dimer, i.e., close to the cell membrane (such a dimer was termed cis-dimer) [6]. This further supports its biological relevance and is discussed in detail in Section 2.3.

For Trop2 ectodomain no existence of a higher-than-dimer oligomeric state has been demonstrated [11], just as for EpCAM [10]. While clustering of Trop2 on apical membranes in polarized cells has recently been reported, there was no evidence of clusters formed by direct interaction bwteen Trop2 molecules. Rather, cluster formation appears to be promoted by local membrane composition (lipid rafts) and is affected by actin depolymerization [39]. Still, to investigate if any of the observed crystal contacts could be reminiscent of a biologically relevant interaction, we examined all possible dimer–dimer contacts (Figure 2). Considering their weakness (assemblies 3–5), incompatibility of their relative orientation with expected position of the anchoring point to transmembrane domain in the juxtaposed membrane (assembly 3), and/or implied polymerization to form a curved assembly (assembly 2) or a zig-zag arrangement (assembly 5), none of them seem to represent a biologically relevant higher-than-dimer oligomer compatible with existing literature data. Therefore, the only biologically relevant situation is represented by assembly 1 corresponding to a dimer formed by two subunits on the surface of the same cell.

#### 2.2.3. General Structural Features of the Trop2 Ectodomain

**Figure 3:**
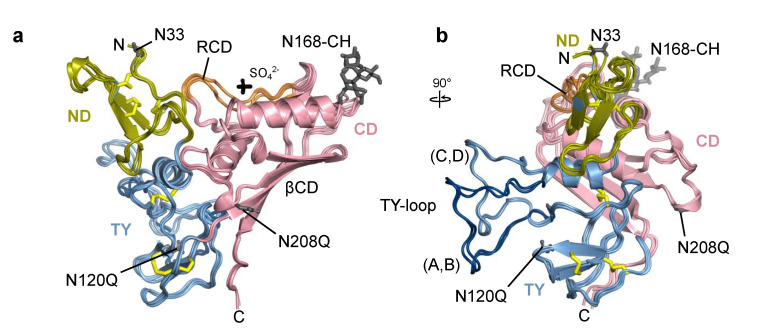
Superposition of individual chains of Trop2 ectodomain from the asymmetric unit shown in two orientations depicting (**a**) triangular domain arrangement, and (**b**) TY-loop protruding from the subunit. Chains are shown in ribbon representation with color-coding corresponding to individual domains (ND in olive, TY in light blue, CD in pink). Disulfide bonds, carbohydrate residues (CH), and sulfate ion (SO_4_^2−^) are shown as yellow, grey, and black sticks, respectively. Unmutated potential N-glycosylation sites (N33, N168) and mutated ones (N120Q, N208Q) are shown as grey sticks. For clarity, individual amino acid and carbohydrate residues and the sulfate ion are shown only for chain A. Ridge region within the CD (RCD) is shown in orange, and the TY-loop of chains A and B in dark blue. Labels N and C denote N- and C-termini, respectively.

All four chains of Trop2 ectodomain located within the asymmetric unit are, in general, structurally very similar. Domains ND (Q31–L69), TY (T70–L148) and CD (V149–T274) form a triangular arrangement where each domain forms contacts with the other two (Figure 3a). The fold of the ND and TY is stabilized by three disulfide bridges with the same cysteine–cysteine connectivity as observed in EpCAM [6,33]. The loop of the TY (residues S81–G102) protrudes from the otherwise compact ectodomain and is (via contacts with concave β-sheet of the CD (βCD)) part of the assembly 1 interface which is in detail described in Section 2.3. Interestingly, the TY-loop adopts two distinct conformations: one in chains A and B, and the other in chains C and D (Figure 3b). The other more pronounced conformational differences are within the RCD region (Q237–R247). Not considering the TY-loop and RCD in structural comparison of pairs of chains, the root-mean-square deviation of C_α_ atoms is in the range from 0.31 Å (chains A and B) to 0.61 Å (chains C and D) with an average value of 0.56 Å.

The N-terminal pyroglutamate and C-terminal residues from F268 (chains A and D) or S269 onwards (chains C and B) were not resolved in electron density map. Two sulfate ions were modelled near the RCD region of chains A and B; the ions are surrounded by terminal parts of side chains of three nearby arginine residues (R178, R183, R239). The SO_4_^2–^ ion likely represents a crystallization artefact since ammonium sulfate was the major precipitant during crystallization. Still, its presence could have affected the local conformation of the RCD which had a certain degree of structural flexibility as indicated by less defined electron density in this region.

Two potential N-glycosylation sites (N33, N168) were left intact in the crystallized protein. Electron density corresponding to the attached carbohydrate moiety was identified only at N168 and modelled as N-acetylglucosamine (NAG) in chains C and D, and as a short branched NAG-[a-(1-6)-FUC]-NAG in chains A and B (GlycoCT notation [40], FUC stands for fucose; Figure S5). This trisaccharide corresponds to the Asn-proximal part of the oligosaccharide often found in insect cells [41], for example in insect cell-produced anti-EpCAM antibody [42]. The utilization of potential N-glycosylation sites in Trop2 in human cells is yet unknown. To compare with EpCAM, only two glycosylation sites are equivalent (Figure 1). In EpCAM from human cells all three N-glycosylation sites are utilized: N74 (within TY-loop, equivalent site not present in Trop2) and N111 (equivalent to N120 in Trop2) are glycosylated to a variable degree, and glycosylation at N198 (N208 in Trop2) is critical for EpCAM stability [43]. On the contrary, in insect cell-produced EpCAM only N74 and N111 were glycosylated while N198 was not [33]. Since the glycosylation pattern of EpCAM in insect and human cells differ, we cannot conclude that in human cells Trop2 is indeed glycosylated at N168. Nevertheless, it is interesting to note that this glycosylation site has no equivalent in EpCAM (Figure 1).

### 2.3. Trop2 Ectodomain Dimer and It’s Implications in Trop2 Proteolytic Cleavage

**Figure 4:**
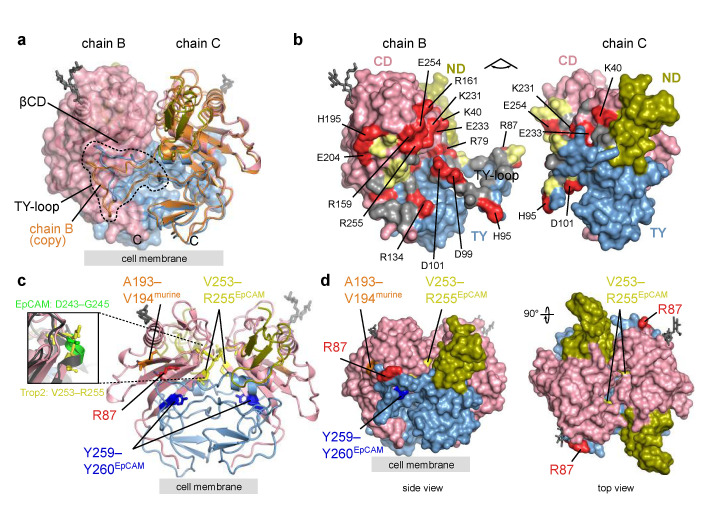
Trop2 ectodomain dimer—alternative TY-loop conformation, interface residues, and location of proteolytic cleavage sites. Domain color-coding is the same as in Figure 3. Carbohydrate residues are shown as grey sticks. Position of the cell membrane (proximal to the C-termini) is denoted by a grey rectangle. (**a**) Assembly composed of chains B and C corresponds to a cis-dimer (Section 2.2.2). Both chains are shown in ribbon representation and for chain B a transparent molecular surface is shown. A copy of chain B superimposed on chain C is shown in orange showing an alternative TY-loop conformation within the same dimer. (**b**) Opened Trop2 ectodomain dimer in surface representation. Charged, polar and hydrophobic residues at the dimer interface are shown in red, yellow and grey. Charged residues are labeled. (**c**) Cleavage sites mapped to Trop2 ectodomain dimer (chains B and C, as in (**a**)). Matriptase and ADAM10 site is shown in red (R87), TACE site equivalent from murine Trop2 in orange (A193–V194), TACE site equivalent from human EpCAM in yellow (V253–R255), and BACE site equivalent from human EpCAM in dark blue (Y259–Y260). Zoomed-in section highlights different conformation of TACE cleavage site in EpCAM (D243–G245, bright green) and the location-equivalent site in Trop2 (V253–R255, yellow). (**d**) The same cleavage sites as in (**c**) shown on surface representation of Trop2 ectodomain dimer in two orientations.

The Trop2 ectodomain dimer identified from crystal contacts as assembly 1 (Section 2.2.2) is stabilized by extensive interactions between the two subunits. A large portion of the dimer interface is formed between TY-loop of one subunit and the concave β-sheet of the juxtaposed CD (βCD; Figure 4a,b). The conformation of TY-loops of the two subunits forming the dimer (chains A and D, or chains B and C) differs (Figure 4a), and the electron density for the TY-loop was not as well defined as for the central part of the molecule. To compare, in another reported EpCAM ectodomain crystal structure the electron density of some sections of the TY-loop was not observed, although EpCAM crystallized as the dimer [7]. This indicates some structural plasticity of the TY-loop region, regardless of the numerous subunit–subunit interactions of charged (salt bridge) and hydrophobic character (Figure 4b), which was also revealed by the contact analysis (Section 2.2.2 and Figure 2c). The Trop2 dimer could be additionally stabilized by dimerization of the transmembrane (TM) helices as previously demonstrated by molecular dynamics simulations of two TM helices corresponding to the Trop2 TM domain embedded in a lipid bilayer [19].

Mapping of proteolytic cleavage sites reported in the literature reveals that all are at least partially accessible in Trop2 dimer (Figure 4c,d). First, the matriptase cleavage site at surface-exposed R87 [20,44] is located within the TY-loop, similarly as the equivalent matriptase cleavage site in EpCAM (R80–R81; Figure 1) [6,45]. Although the TY-loop is part of the dimer interface, the structural plasticity described above may (via temporal TY-loop disengagement from juxtaposed βCD) enable efficient cleavage by enhancing the accessibility of the cleavage site. Interestingly, it was shown that mutation V294A (part of the βCD, shown in orange in Figure 4c) prevents matriptase cleavage at R87 without affecting the Trop2 dimerization, and the observed effect was attributed to impaired binding of matriptase to the Trop2 as the substrate [44]. Since in our crystal structure side chains of V194 and the nearby V191 are both located on the opposite side of βCD than the juxtaposed TY-loop and are therefore not exposed, it is likely that side chain of V194 is not directly involved in matriptase interaction. It is more likely that the mutation V194A affects local conformation of the cognate β-strand and thereby influences matriptase binding. Bond R87—T88 was also identified as the cleavage site for ADAM10 [24], and the same accessibility principles may apply as described above. Both matriptase and ADAM10 cleavages result in a nicked polypeptide chain where the N-terminal fragment (ND plus first part of TY) is still anchored to the rest of the subunit via the first disulfide linkage within the TY (C73–C108).

Next, Trop2 is cleaved by TACE/ADAM17, since murine Trop2 cleavage at A187–V188 has been identified [15], which translates via amino acid sequence alignment to A193–V194 in human Trop2 (Figure 4c). This site is accessible in Trop2 dimer (Figure 4c,d) and involves the V194 residue important for matriptase-mediated cleavage at R87 as discussed above. In EpCAM the location-equivalent site would be I184–L185. However no cleavage at this site has been demonstrated up to now. On the contrary, TACE cleavage site in EpCAM (D243–G245) is completely buried in the dimer [8,13] as depicted by the mapped location-equivalent V253–255 site in Trop2 (Figure 4c,d). Also, the local conformation of these two location-equivalent sites is markedly different. In EpCAM, this site is a part of a short helix while the corresponding region in Trop2 forms a loop region at the start of β-strand within βCD (Figure 4c, enlarged area on the left). The other cleavage site in EpCAM where cleavage is mediated by BACE (Y250–Y251) and most probably happens upon dimer dissociation in acidic conditions [12,13] is perfectly preserved in Trop2 (Y259–Y260; Figure 4c). However, cleavage of Trop2 at this site has not yet been demonstrated.

### 2.4. Trop2 and EpCAM Significantly Differ in Their Membrane-Distal Regions

**Figure 5:**
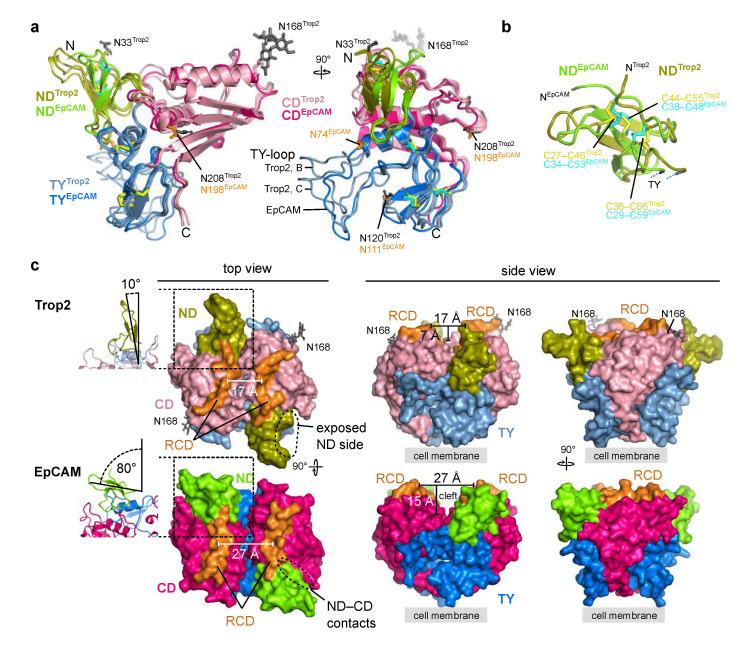
Comparison of Trop2 and EpCAM ectodomain subunit structures and dimers. As EpCAM structure PDB ID 4MZV was used [6]. Trop2 domain color-coding is the same as in Figure 3, and ND, TY and CD domains of EpCAM are shown as chartreuse, marine blue and dark pink, respectively. RCD region (H227–Q239 in EpCAM, Q237–R247 in Trop2) is shown in orange. (**a**) Superposition of two chains of Trop2 ectodomain (chains B and C) with EpCAM ectodomain structure (PDB ID 4MZV) shown in two orientations depicts different conformations of the TY-loop. Disulfide bridges are shown as sticks (yellow for Trop2, cyan for EpCAM). (**b**) Superposition of ND of Trop2 (olive) and EpCAM (chartreuse) shows conserved pattern of three closely located disulfide bridges (color-coded the same as in (**a**)). (**c**) Surface representation of Trop2 and EpCAM ectodomain dimer in three different orientations demonstrates significant difference in relative orientation of the ND, different position of the RCD and a much narrower cleft in the membrane-distal region of Trop2 as compared to EpCAM. Relative orientation of the ND was defined as the angle between β-sheet of the ND and the dimer interface plain (left, ribbon representation of the region marked by rectangle). Cleft width was defined as the length of the vector (parallel to the membrane) between central parts of the juxtaposed RCDs, and cleft depth as the length of the vector (perpendicular to the membrane) between the most membrane-distal part of the dimer and the touching point of the subunits in the middle part of the dimer.

Considering high amino acid sequence similarity between Trop2 and EpCAM (Figure 1) the structures are likewise expected to be similar. Indeed, the overall domain arrangement and the fold of individual domains is mostly the same (Figure 5a). One of the more significant differences is the conformation of the TY-loop (Figure 5a). Also, in Trop2 two N-glycosylation sites are located within the membrane-distal region (N33 and N168; Figure 5a), however equivalent sites are absent in EpCAM. On the contrary, all three N-glycosylation sites in EpCAM are located at the lateral side of the molecule. It was hypothesized that they help in correct orientation of the dimer relative to the membrane [6].

The arrangement of the three closely spaced disulfide bridges that stabilize the fold of the ND is the same in Trop2 and EpCAM (Figure 5b). However, relative orientation of the short β-strands is different (Figure 5b). Interestingly, the ND is the most immunogenic domain of EpCAM and is targeted by the vast majority of the anti-EpCAM antibodies [6]. ND is also the domain that is the most different between Trop2 and EpCAM—the amino acid sequence percentage identities are 33% for ND, 58% for TY and 47% for CD. Therefore, ND-targetting antibody cross-reactivity seems unlikely.

Described differences between ND of Trop2 and EpCAM translate to the different relative orientation of this domain with regard to the rest of the molecule (Figure 5c). The angle defined by the β-sheet and the dimer interface plain is in Trop2 very narrow (10°) and the ND is almost colinear with the plain. However, in EpCAM the orientation of the ND with regard to this plain is almost perpendicular (angle of 80°; Figure 5c, left). In line with this, the lateral contacts between the ND and CD are in EpCAM more extensive (Figure 5c, left). Therefore, the positioning of ND relatively the rest of the subunit/dimer is in Trop2 less compact with a higher degree of lateral accessibility than in EpCAM. Also, the ridge-of-CD (RCD) region is markedly different. In Trop2 it is curved and the together with the nearby regions almost completely closes the inter-subunit cleft (Figure 5c, middle). In EpCAM this cleft is much more pronounced, both in terms of width and depth (59% broader and 114% deeper as in the Trop2). These significant differences at this most exposed membrane-distal part of the dimer could underly the distinct interactome of Trop2 (IGF-1, neuregulin-1, α_5_β_1_ integrin) and EpCAM (EGFR), which in turn translates into their functional differences as outlined in the introduction.

In addition to significant structural differences within the membrane-distal regions of Trop2 and EpCAM also the dimer stability appears to be different. Equivalent interface analysis as for Trop2 ectodomain dimer (assembly 1 in Figure 2c) shows that the dimer interface in EpCAM is smaller (1987 Å^2^ compared to 2474 Å^2^ for Trop2) and more polar (Δ^i^G of –8.5 kcal/mol compared to –18.2 kcal/mol for Trop2). In EpCAM the interaction is mediated by 16 salt bridges compared to nine salt bridges in Trop2, and a triple TY-loop mutant of EpCAM ectodomain (K83D, P84D, L88D) was found to be constitutively monomeric [14]. No comparable mutations were yet reported for Trop2 dimer destabilization, however single alanine mutations (R87A within TY-loop, and K189A and H195A within βCD) did not affect alter Trop2 oligomeric state [44]. Still, since the latter mutations were introduced into the full-length protein direct comparison with isolated ectodomains is probably not possible due to different molecular context (additional dimer stabilization via TM dimerization as described above).

Interestingly, a tighter Trop2 dimer and different position of the α-site for proteolytic cleavage by TACE (Section 2.3) could be connected to a different cleavage mechanism. As noted, in EpCAM dimer dissociation/destabilization is needed to reveal the α-site, while in the tighter Trop2 dimer the α-site is accessible even in the dimeric form. Therefore, molecular events leading to cleavage may be significantly different.

While the described differences may be a consequence of different crystal packing found in Trop2 and EpCAM crystals, analysis of available structural data (one Trop2 and two EpCAM ectodomain structures) indicate that this is less likely. First, the four chains making up the two dimers in the asymmetric unit of Trop2 crystal have different inter-molecular contacts, however the conformation of the membrane-distal region and relative position of the ND is similar (Figure S6). Second, comparison of two EpCAM ectodomain structures, one of EpCAM with a bound small unnatural ligand (PDB ID 4MZV [6]), and the other of EpCAM in complex with a single-chain variable fragment bound to the ND (PDB ID 6I07 [7]), demonstrates that the markedly different crystal and inter-molecular contacts do not significantly affect the relative position of the ND nor the conformation of the membrane-distal region (Figure S6).

## 3. Materials and Methods

### 3.1. Expression and Purification of Trop2 Ectodomain for Crystallization

Protein for crystallization (mutant Trop2 ectodomain with N120Q and N208Q mutations) was prepared in insect cells using Bac-to-Bac system (Life Technologies, Carlsbad, USA). As the base sequence, human Trop2 cDNA clone ID IRALp962I2113Q was used (Source BioScience imaGenes, Berlin, Germany). First, the plasmid construct based on pFastBac1 vector was prepared harboring the sequence coding for extracellular part of human Trop2 with the wild-type signal sequence (UniProt P09758-1, residues 1–274) which was fused to a His_6_-tag-coding sequence (at 3′-end). Two glycosylation-abolishing mutations (N120Q, N208Q) were introduced via method of two-sided splicing by overlap extension using appropriately designed oligonucleotide primers [46]. This enabled higher protein sample homogeneity than is possible with a completely wild-type protein due to heterogeneous glycosylation in insect cell expression system. At the same time, solubility problem associated with the mutant protein with all four N-glycosylation sites mutated to Q was overcome [11]. Recombinant bacmid was prepared using Bac-to-Bac system protocol, however *E. coli* DH10MultiBac cells were used instead of DH10Bac cells to prevent extensive protein degradation due to V-CATH protease encoded in the wild-type baculoviral genome [11]. To produce recombinant baculoviruses, *Spodoptera frugiperda* Sf9 insect cells (Novagen, Madison, Wisconsin, USA) were transfected with recombinant bacmid using TurboFect transfection reagent (Thermo Scientific, USA). Cell culture supernatant was pooled 3 days post infection and used in two sequential amplification cycles to produce high-titer baculoviral stock, which was then used to infect larger volume (2 L) of Sf9 cell culture at a density of 2 × 10^6^ cells mL^−1^ and at a multiplicity of infection of 10. Cell cuture supernatant was harvested 3 days post infection by centrifugation at 1000× *g* for 10 min. To the supernatant 1.5 M Tris-HCl, pH 8.0, was added to reach the pH of 8.0, and the solution was mixed for 30 min at 4 °C. Precipitated substances were removed by another centrifugation step (10,000× *g* for 20 min).

From cleared culture supernatant the Trop2 ectodomain was purified by two sequential immobilized metal affinity chromatography (IMAC) steps followed by size exclusion chromatography (SEC), all performed at room temperature on an ÄKTA FPLC system (GE Healthcare, Chicago, Illinois, USA). For IMAC, a 5 mL Ni^2+^-loaded cOmplete His-Tag purification column (Roche, Switzerland) was used, equillibrated in binding buffer (20 mM Tris-HCl, pH 8.0, 400 mM NaCl); proteins were eluted using 20 mM Tris-HCl, pH 8.0, 200 mM NaCl, 500 mM imidazole. Between the two IMAC steps imidazole was removed by dialysis against 20 mM Tris-HCl, pH 8.0, 100 mM NaCl. For final SEC purification step a Superdex 200 10/300 GL column (GE Healthcare, Chicago, IL, USA) was used, equilibrated in 20 mM Na^+^-HEPES, pH 8.0, 100 mM NaCl. Protein was concentrated using Amicon centrifugal concentrators with 3 kDa molecular weight cut-off (Merck Millipore, Burlington, MA, USA) to a final concentration of 15.8 mg/mL (determined via measurement of absorbance at the wavelength of 280 nm).

Purity of the final protein sample was verified by polyacryl gel electrophoresis in the presence of sodium dodecylsulfate (SDS-PAGE) under reducing and non-reducing conditions (Figure S1).

### 3.2. N-Terminal Sequencing and Sequence Analysis

Protein sample for N-terminal sequencing was prepared by reverse-phase chromatography using a VYDAC Protein C4 column (Grace Davison Discovery Sciences, Deerfield, IL, USA) on an Agilent Technologies Series 1200 HPLC (USA). 100 µg of Trop2 ectodomain (N120Q, N208Q) was loaded. During binding and washing 0.1% (*v*/*v*) trifluoroacetic acid (TFA) was used, and the bound proteins were eluted with a gradient of elution solvent (0.1% (*v*/*v*) TFA, 90% (*v*/*v*) acetonitrile) (Figure S2). The single peak was collected and dried. N-terminal sequencing was performed by Proteome Factory AG (Berlin, Germany) as a two-step procedure: (1) deblocking the N-terminus, and (2) Edman amino acid sequencing. Briefly, a 500 pmol protein aliquot was blotted onto a PVDF membrane, blocked with polyvinylpolypyrrolidone (PVPP), 3× washed with water and incubated with *Pfu* Pyroglutamate Aminopeptidase (TaKaRa Bio, Göteborg, Sweden) for 1 h at 80 °C. The membrane was then 3× washed with water, dried by argon airflow, and used in an ABI Procise sequencer (Applied Biosystems, Waltham, MA, USA). Here, five N-terminal amino acid sequencing steps were performed using the Procise method “puls liquid”. The chromatograms of N-terminal sequence analysis were manually evaluated in respect to retention times of the phenylthiohydantoin (PTH) amino acids of interest, and changes in peak intensities (Figure S3).

Signal peptide cleavage site was predicted using SignalP 5.0 with settings adjusted for Eukarya (Figure S4) [25]. For global pairwise sequence alignment the EMBOSS Needle software was used [47].

### 3.3. Crystallization

Crystals were grown using the sitting drop vapor-diffusion method. Reservoir solution (30 µL) was composed of 1.6 M ammonium sulfate, 0.5 M NaCl, 13.3 mM EDTA, 0.1 M MES pH 5.8. Drops were composed of 1 µL of reservoir solution and 1 µL of purified Trop2 ectodomain (N120Q, N208Q; in 20 mM Na^+^-HEPES, 100 mM NaCl, pH 8.0) at the protein concentration of 10 mg/mL. Crystals were mounted in cryo-loops, briefly soaked in cryo-solution (the same composition as the reservoir solution with added 20% (*v*/*v*) glycerol), and flash-frozen in liquid nitrogen.

### 3.4. Data Collection and Structure Solving

Diffraction data were collected at the XRD2 beamline of the Elettra Synchrotron, Basovizza, Italy, equipped with the Dectris Pilatus 6M detector. Data were collected at the temperature of 100 K, oscillation angle was 0.5°. Other experimental parameters are listed in Table 1. Diffraction data were processed using XDS Program Package [48], which was followed by symmetry determination using POINTLESS [49] and scaling using AIMLESS [50]. Phasing was done by molecular replacement in Phaser [51] using the crystal structure of EpCAM ectodomain (PDB ID 4MZV) [6] as a model. This model was modified by removing the N-terminal domain and the long loop of the thyroglobulin domain. Only four copies of the Trop2 ectodomain were located within the asymmetric unit giving a high solvent content of 72%. Considering the usual solvent content of protein crystals in the range 40–60%, the number of copies expected would be six to eight [52,53]. Electron density corresponding to one ectodomain copy (chain D) was less defined as for the other three copies, most probably because this copy is not involved in as many crystal contacts as the others (Figure 2 and Figure S6). After automatic rebuilding using BUCCANEER [54,55], the final structural model was produced after several cycles of manual rebuilding in Coot [56] and automatic refinement using Phaser [51] as part of the PHENIX 1.19.2 software package [57].

### 3.5. Structure Analysis

Crystal contacts/interfaces were analyzed using “Protein interfaces, surfaces and assemblies” service PISA at the European Bioinformatics Institute (http://www.ebi.ac.uk/pdbe/prot_int/pistart.html, accessed on 13 August 2021) [38]. Structure figures were prepared using PyMOL [58]. Structure alignments were calculated using CEAlign [59].

## 4. Conclusions

The crystal structure described in the article is the first experimentally determined structure of Trop2. Crystal contact analysis revealed a tight assembly composed of two Trop2 subunits which, by analogy to homologous EpCAM ectodomain dimer, represents a biologically relevant dimer. While the general fold of Trop2 ectodomain is very similar to the fold of EpCAM, there are several structural differences. The highly immunogenic N-terminal domain (ND) is laterally more exposed in Trop2 than in EpCAM, and the membrane-distal region of Trop2 dimer is much tighter. At the equivalent position in EpCAM a pronounced inter-subunit cleft is present, while in Trop2 dimer this cleft is virtually absent. Furthermore, the proteolytic cleavage site for TACE within Trop2 ectodomain maps to the surface-exposed edge of a β-strand, while in EpCAM it is part of a short α-helix and buried in the dimer interface. The uncovered structural differences are likely connected to their distinct interactome and indicate different mechanism of the initial signaling-associated proteolytic cleavage event.

## Figures and Tables

**Table 1:** Data collection and refinement statistics for the crystal structure of Trop2 ectodomain.

PDB ID	7PEE
**Data collection**	
X-ray source and beamline	Elettra Synchrotron, XRD2 (11.2C)
Wavelength (Å)	0.9789
Space group	P4_3_22
**Cell dimensions**	
a, b, c (Å)	145.08, 145.08, 217.77
α, β, γ (°)	90, 90, 90
**Data statistics**	
Resolution range (Å) ^a^	48.36–2.81 (2.91–2.81)
Total no. of reflections ^a^	376,492 (36,911)
No. of unique reflections ^a^	57,144 (5604)
Mean I/σ(I) ^a^	15.27 (1.73)
*R*_merge_ (%) ^a,b^	11.88 (127.2)
CC_1/2_ ^a,b^	0.997 (0.692)
Completeness (%) ^a^	99.59 (99.48)
Redundancy ^a^	6.6 (6.6)
**Number of atoms**	
Total	7620
Protein/Water/Ligands	7476/30/114
**Refinement statistics**	
*R*_work_/*R*_free_ (%) ^c^	23.94 (26.30)
*Root-mean-square deviations*	
Bond lengths (Å)	0.007
Bond angles (°)	1.07
*Ramachandran plot*	
Favored/Allowed/Outliers (%) ^d^	95.75/4.25/0.00
Rotamer outliers (%) ^d^	1.71
*B-factor*	
Average	75.80
Protein/Ligands/Solvent	75.12/125.23/57.60

^a^ Values in parentheses refer to the highest resolution shell. ^b^ $R_{merge}=\sum_{hkl}\sum_{i}\left|I_{i}\left(hkl\right)-\langle I\left(hkl\right)\rangle \right|\;/\;\sum_{hkl}\sum_{i}\left|I_{i}\left(hkl\right)\right|$, where 〈I(hkl)〉 is the mean insensity of a set of equivalent reflections. ^c^  $R_{work}=\sum_{hkl}|\left|F_{obs}\right|-\left|F_{calc}\right||\;/\;\sum_{hkl}\left|F_{obs}\right|$, where $F_{obs}$ and $F_{calc}$ are observed and calculated structure factors, respectively. $R_{free}$ was calculated in the same way as $R_{work}$, however only a test set consisting of 5% of data excluded from refinement calculation was used. ^d^ Values were obtained using MolProbity [34].

## Data Availability

Structure of Trop2 ectodomain and the accompanying data (processed diffraction data, electron density maps) are deposited in the Protein Data Bank under the accession code 7PEE (DOI 10.2210/pdb7PEE/pdb).

## Supplementary Materials

The following are available online at https://www.mdpi.com/article/10.3390/ijms221910640/s1. Figure S1: SDS-PAGE analysis of Trop2 ectodomain protein sample and photo of the crystals obtained; Figure S2: Analysis of final protein sample (Trop2 ectodomain) on reverse-phase chromatography; Figure S3: Chromatograms obtained during N-terminal sequencing after deblocking (removal of pyroglutamate); Figure S4: Prediction of the wild-type signal peptide cleavage site of human Trop2 (UniProt P09758-1) using the SignalP 5.0 server; Figure S5: Electron density at N168 for each of the four chains labeled A to D in the asymmetric unit; Figure S6: Comparison of dimer and subunit structures, and analysis of crystal contacts in the Trop2 ectodomain dimer structure reported in this manuscript (PDB ID 7PEE), and in the two EpCAM ectodomain structures (PDB ID 4MZV and 6I07).
