# Supplementary material for: Trop2 Forms a Stable Dimer with Significant Structural Differences within the Membrane-Distal Region as Compared to EpCAM
Source: Int J Mol Sci. 2021 Sep 30;22(19):10640. doi: 10.3390/ijms221910640 (PMC8508679; doi:10.3390/ijms221910640)
Supplement: Supplementary file 1 [file ijms-22-10640-s001.zip › ijms-1362493-supplementary.pdf]

SUPPLEMENTARY MATERIALS

Trop2 Forms a Stable Dimer with Significant Structural Differences within the Membrane-Distal Region as Compared to EpCAM

Miha Pavšič

Department of Chemistry and Biochemistry, Faculty of Chemistry and Chemical Technology, University of Ljubljana, Večna pot 113, SI-1000 Ljubljana, Slovenia; miha.pavsic@fkkt.uni-lj.si; Tel.: +386-1-479-8550

Protein sample characterization and crystallization

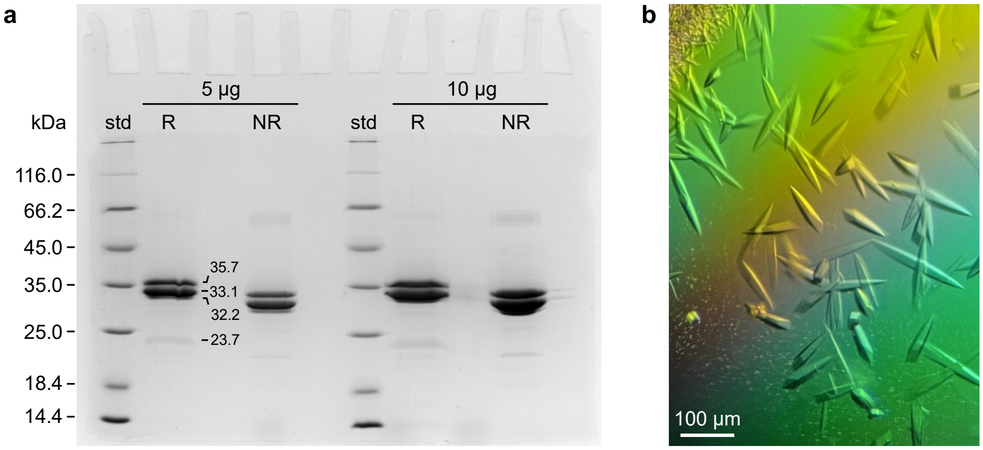

Figure S1. SDS-PAGE analysis of Trop2 ectodomain protein sample and photo of the crystals obtained. (**a**) SDS-PAGE analysis of purified Trop2 ectodomain (R and NR denote reducing and non-reducing conditions, respectively). Sample was loaded as a 5 and 10 µl aliquot. Gel was stained using Coomassie Brilliant Blue. The apparent molecular weight (M_w; calculated using ImageLab 6.0.1 software (Bio-Rad, U. S. A.)) of major species is in the range of 33–36 kDa. This is higher than the M_w calculated from amino acid sequence (28.3 kDa, considering signal peptide cleavage at just before Q30), and higher than the apparent M_w of the completely non-glycosylated form determined using the same approach (32 kDa) [1]. This M_w difference and several bands on SDS-PAGE for the double mutant indicate heterogeneous glycosylation. (**b**) Crystals of Trop2 ectodomain.

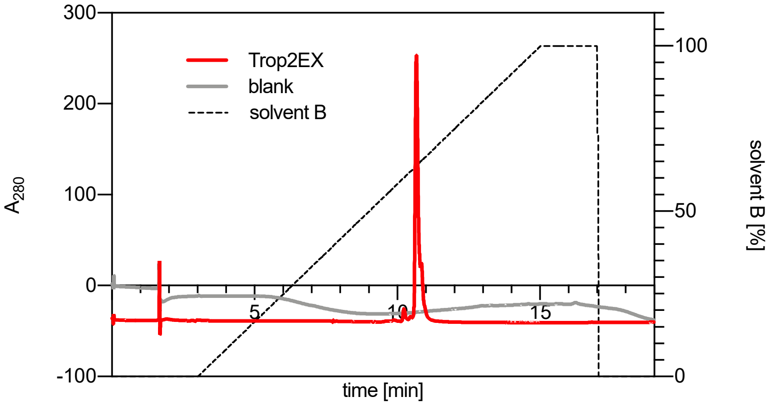

Figure S2. Analysis of final protein sample (Trop2 ectodomain) on reverse-phase chromatography. The single peak was collected and used for N-terminal sequencing. Solvent B corresponds to 0.1% (v/v) TFA, 90% (v/v) acetonitrile.

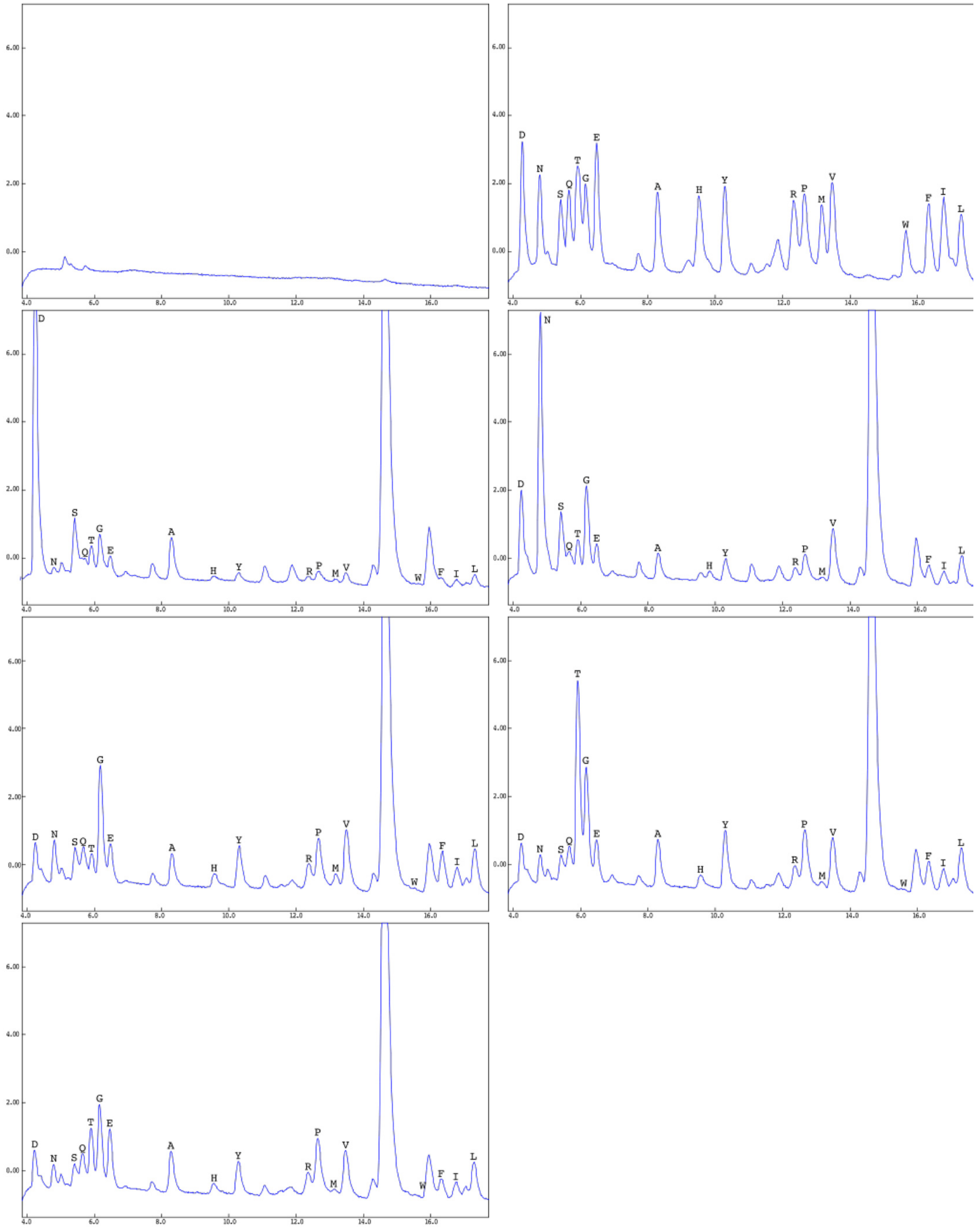

Figure S3. Chromatograms obtained during N-terminal sequencing after deblocking (removal of pyroglutamate). Cycle numbers correspond to numbering of sequential N-terminal amino acid residues starting with 1 from the mature Trop2 ectodomain. The determined sequence was DNXTX where X corresponds to no amino acid residue detected, most probably due to the presence of cysteine residue at this site. The aspartate in the determined sequence is preceded by pyroglutamate (modified Q31) which was removed during deblocking procedure. Single N-terminal sequence indicates uniform signal peptide processing and absence of proteolytic cleavage at other sites.

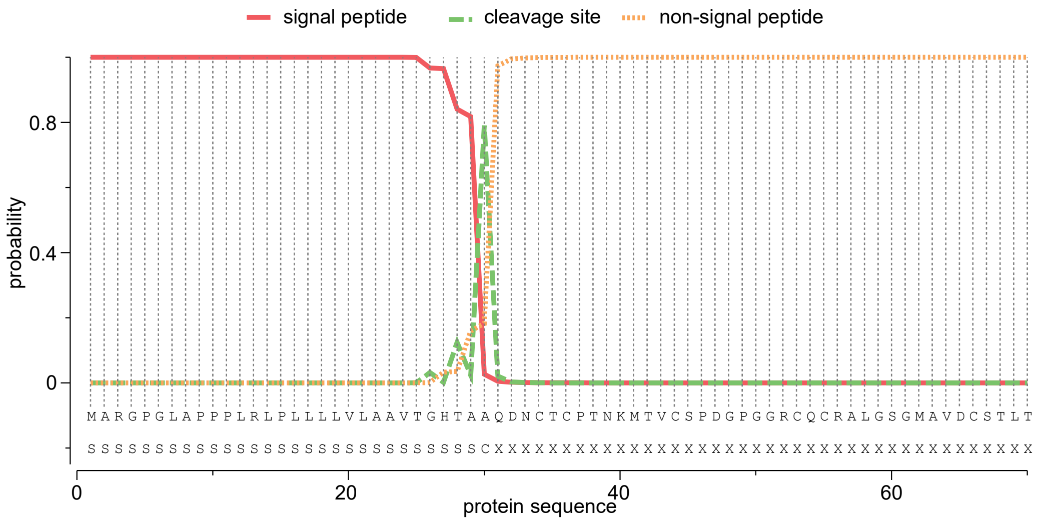

Figure S4. Prediction of the wild-type signal peptide cleavage site of human Trop2 (UniProt P09758-1) using the SignalP 5.0 server (http://www.cbs.dtu.dk/services/SignalP/) [2]. As organism group Eukarya was used. For each amino acid residue of the first 70 residues three probability scores have been assigned—being part of signal peptide (S) or other part of sequence (non-signal peptide, X), or representing the cleavage site (C). The classification of each residue based on highest probability is printed below the amino acid sequence. Analysis indicates that signal peptide cleavage most likely occurs at Q31.

Structure determination

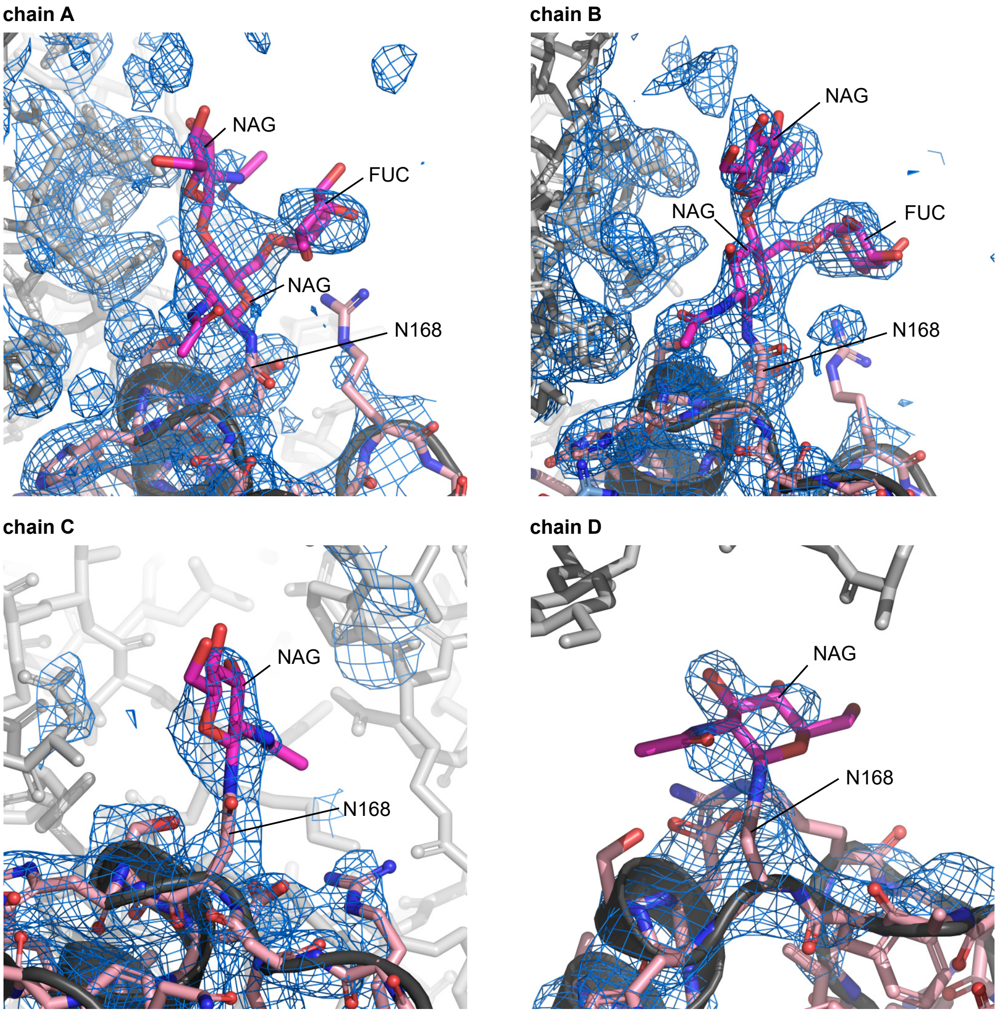

Figure S5. Electron density at N168 for each of the four chains labeled A to D in the asymmetric unit. 2Fo-Fc electron density map is shown at 1σ contouring level as a blue mesh. Density is shown only for N186 and carbohydrate moiety plus region within 8 Å distance. NAG and FUC denote N-acetylglucosamine and fucose, respectively. Molecules are shown in stick representation—in one asymmetric unit color-coded by atom type (protein carbon atoms in light pink, carbohydrate carbon atoms in magenta, and oxygen and nitrogen atoms in red and blue, respectively), and in the adjacent asymmetric units as light grey sticks. For the asymmetric unit in focus the polypeptide chain is additionally shown as dark grey ribbon.

Analysis of crystal contacts

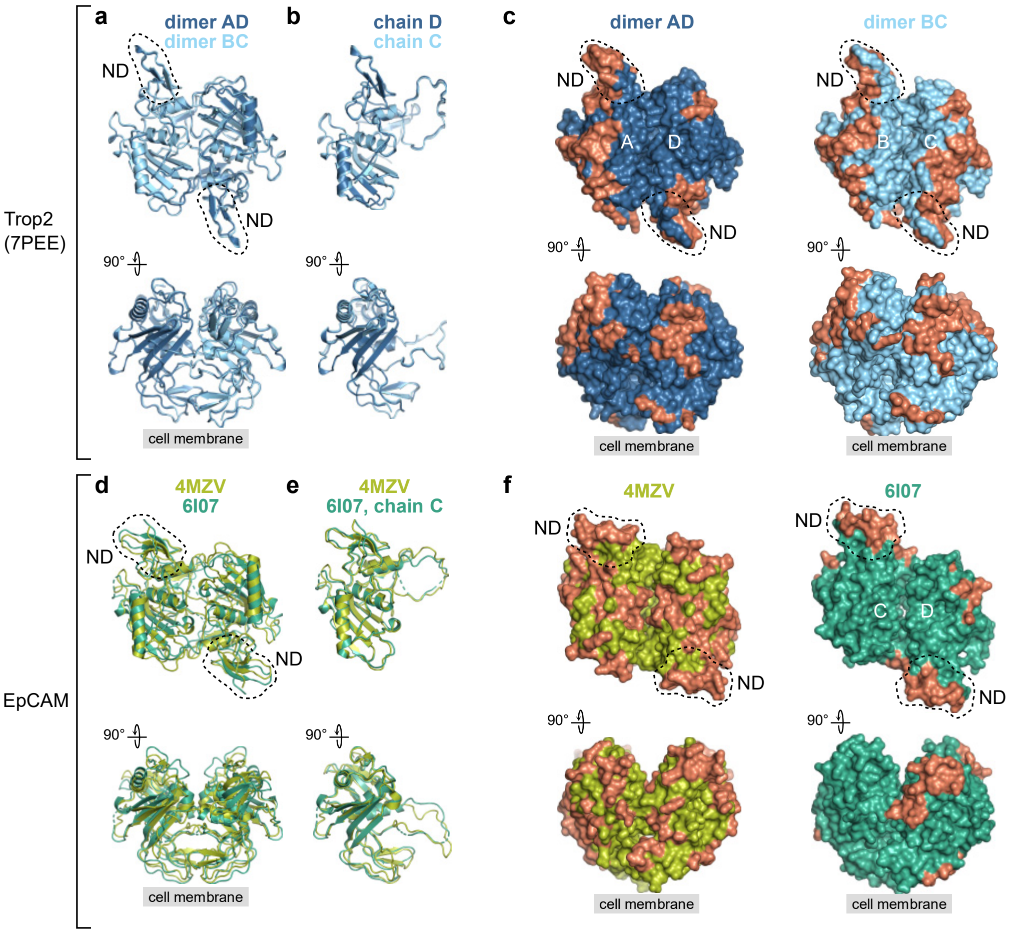

Figure S6. Comparison of dimer and subunit structures, and analysis of crystal contacts in the Trop2 ectodomain dimer structure reported in this manuscript (PDB ID 7PEE), and in the two EpCAM ectodomain structures (PDB ID 4MZV [3] and 6I07 [4]). (**a**) Superposition of the two dimers (one composed of chains A and D, the other of chains B and C) of Trop2 ectodomain from the same asymmetric unit and (**b**) of the chains C and D from separate dimers from the same asymmetric unit shows an almost identical polypeptide chain conformation, and identical relative subunit and domain orientation despite different local environment as depicted in (**c**) where residues involved in inter-dimer contacts are colored orange. (**d**) Superposition of two crystal structures of EpCAM ectodomain dimer displays some differences in relative subunit orientation, while (**e**) the superposition of subunit structures reveals high overall similarity (discussed/reviewed in [5]), despite (**f**) markedly different crystal and ligand contacts (orange). The structure deposited under PDB ID 6I07 is of EpCAM ectodomain in complex with a single-chain variable fragment of the anti-EpCAM MM-131 antibody bound to the ND, while the structure under PDB ID 4MZV contains only the EpCAM ectodomain with bound n-decyl-β-D-maltopyranoside. Residues involved in crystal and other inter-molecular contacts with the dimer shown were defined as those that are within 5 Å distance from any other protein or ligand atoms in the crystal (except water molecules). Interface area and Δ^iG for the dimer in PDB ID 4MZV are 2438 Å^2 and −11.4 kcal/mol, respectively, and for the PDB ID 6I07 1243 Å^2 and −9.6 kcal/mol, respectively (calculated using PDBePISA [6]). The small interface area in 6I07 reflects the lack of coordinates for parts of the TY-loop (electron density not well-defined), which is also reflected in visual difference of this dimer from 4MZV as shown in upper part of panel (**f**). ND marks the N-terminal domain of Trop2/EpCAM.

References

1. Vidmar, T.; Pavšič, M.; Lenarčič, B. Biochemical and Preliminary X-Ray Characterization of the Tumor-Associated Calcium Signal Transducer 2 (Trop2) Ectodomain. *Protein Expr. Purif.* **2013**, *91*, 69–76, doi:10.1016/j.pep.2013.07.006.
2. Almagro Armenteros, J.J.; Tsirigos, K.D.; Sønderby, C.K.; Petersen, T.N.; Winther, O.; Brunak, S.; von Heijne, G.; Nielsen, H. SignalP 5.0 Improves Signal Peptide Predictions Using Deep Neural Networks. *Nat. Biotechnol.* **2019**, *37*, 420–423, doi:10.1038/s41587-019-0036-z.
3. Pavšič, M.; Gunčar, G.; Djinović-Carugo, K.; Lenarčič, B. Crystal Structure and Its Bearing towards an Understanding of Key Biological Functions of EpCAM. *Nat. Commun.* **2014**, *5*, 4764, doi:10.1038/ncomms5764.
4. Casaletto, J.B.; Geddie, M.L.; Abu-Yousif, A.O.; Masson, K.; Fulgham, A.; Boudot, A.; Maiwald, T.; Kearns, J.D.; Kohli, N.; Su, S.; et al. MM-131, a Bispecific Anti-Met/EpCAM MAb, Inhibits HGF-Dependent and HGF-Independent Met Signaling through Concurrent Binding to EpCAM. *Proc. Natl. Acad. Sci. U. S. A.* **2019**, doi:10.1073/pnas.1819085116.
5. Gaber, A.; Lenarčič, B.; Pavšič, M. Current View on EpCAM Structural Biology. *Cells* **2020**, *9*, 1361, doi:10.3390/cells9061361.
6. Krissinel, E.; Henrick, K. Inference of Macromolecular Assemblies from Crystalline State. *J. Mol. Biol.* **2007**, *372*, 774–797, doi:10.1016/j.jmb.2007.05.022.
